# The viral and inflammation hypothesis of epileptic seizures based on bioinformatic study of circulating miRNAs and peripheral whole-blood mRNAs of adult epilepsy patients

**DOI:** 10.3389/fneur.2022.909142

**Published:** 2022-09-12

**Authors:** Jiahuan Wu, Ying Meng, Fei Xu, Qian Wu, Cheng Wang

**Affiliations:** ^1^Department of Rehabilitation Medicine, Suzhou Science and Technology Town Hospital, Gusu School, Nanjing Medical University, Suzhou, China; ^2^The Department of Oral and Maxillofacial Surgery, The Affiliated Stomatology Hospital of Nanjing Medical University, Nanjing, China; ^3^Department of Pharmacology, The Affiliated Suzhou Hospital of Nanjing Medical University, Suzhou, China; ^4^Department of Neurology, The First Affiliated Hospital of Nanjing Medical University, Nanjing, China

**Keywords:** epileptic seizure, circulating microRNA, mRNA, bioinformatic analysis, epilepsy

## Abstract

**Background:**

The study aimed to investigate the genome-wide biological significance of the circulating miRNAs markers found in peripheral whole blood of adult epileptic seizures patients by integrating analysis using bioinformatics approaches.

**Methods:**

The Gene Expression Omnibus (GEO) dataset was accessed to retrieve epilepsy-related circulating miRNA profile data (GSE114847) including 89 subjects (*n* = 40 epileptic and *n* = 49 healthy control), peripheral whole-blood mRNA expression data (GSE143772) including 64 subjects (*n* = 32 epileptic and *n* = 32 healthy control). To eliminate age disparities in epilepsy pathophysiology only adult epileptic patients were selected. Furthermore, GEO2R was used to identify adult-related mRNAs (AD-mRNAs) against epilepsy as potential biomarkers. Moreover, to predict the potential target genes for these mRNAs, we used mirWalk. Finally, the Gene Ontology (GO) and Kyoto Encyclopedia of Genes and Genomes (KEGG) were utilized to investigate the biological activities of AD-mRNAs. Importantly, the protein–protein network of these identified AD-mRNAs was constructed. Eventually, the overlapping AD-mRNAs and AD-miRNAs and their functions were explored to shortlist potential AD-epileptic markers.

**Result:**

The current study resulted in the identification of 79 upregulated and 40 downregulated different expression gene (DEGs) in both applied data. These targets were cross-linked and mapped with each other to acquire common adult epilepsy-related overlapped mRNAs (Mo-mRNAs). It was found that there was a total of 36 overlapping genes. These overlapped AD-mRNAs markers were found to be functionally enriched in cell regulating pathways i.e., positive regulation of type 1 interferon signaling pathway and mitochondrial cytochrome C release pathway, respectively.

**Conclusion:**

This research gives a comprehensive depiction of the mRNAs that may be involved in adult epilepsy patients' pathophysiological progressions.

## Introduction

Epilepsy is a complicated neurological disease that exhibits high heterogeneity characterized by the type of seizure that results from the abnormal and synchronous firing of neurons in the brain, the events that occurred immediately before the seizure onset and clinical studies, such as patterns of brain electrical activity, neuroimaging, and biochemical profiling. Nearly, 69 million people are affected by epilepsy around the globe (1). Epileptic seizures cause dynamic, reversible changes in brain function, and are often associated with loss of consciousness (2). Studies have found changes in miRNA levels in the hippocampus of patients with temporal lobe epilepsy and in neural tissues from animal models of epilepsy. Blood of patients with epilepsy has shown differences in the quantity of circulating miRNAs, implying diagnostic biomarker potential (3). MicroRNAs (miRNAs) and target messenger RNAs (mRNAs) are primarily involved in pathological processes such as inflammation, excitotoxicity, apoptosis, and angiogenesis pathways (4). Significantly, the majority of miRNAs are found inside the cell, certain miRNAs, also known as extracellular or circulating miRNAs, have been discovered outside the cell recently (5). Multiple disorders, such as cancer, heart disease, and epilepsy, may be diagnosed using these circulating miRNAs since they can be readily identified in peripheral blood (6). MiRNAs in circulation were previously assumed to have no biological role, according to prior reports. The biological consequences of these biomarkers, however, have only been recently discovered, and it has been suggested that circulating miRNAs may play an essential role in disease development, such as immunoregulation and intercellular signal transmission (7, 8). It is possible to explore gene expression parallels between the blood and the brain since they share several biological pathways. Whole-blood components, such as exosomes, platelets, and leukocytes, play an important role in the pathophysiology of seizures (9–11). Therefore, this research aimed to examine the association between peripheral whole-blood mRNAs and miRNAs in epileptic seizures and to investigate the potential roles of circulating miRNAs in peripheral whole blood of adult epilepsy patients.

The genome-wide investigations may give more information about disease pathophysiology (12). In peripheral blood for epilepsy, genome-wide expression analysis of mRNAs and miRNAs has previously been performed, and putative biological roles in *Homo sapiens* have been hypothesized independently (13). The interconnections between these miRNAs and mRNAs, however, are not well-understood. Furthermore, it is reported that the expression of mRNA was also impacted by age variations in epilepsy cases (14). As a consequence, studies evaluating people in various stages of aging may provide conflicting results. Thus, we solely analyzed data from adult patients in our research.

**Figure 1:**
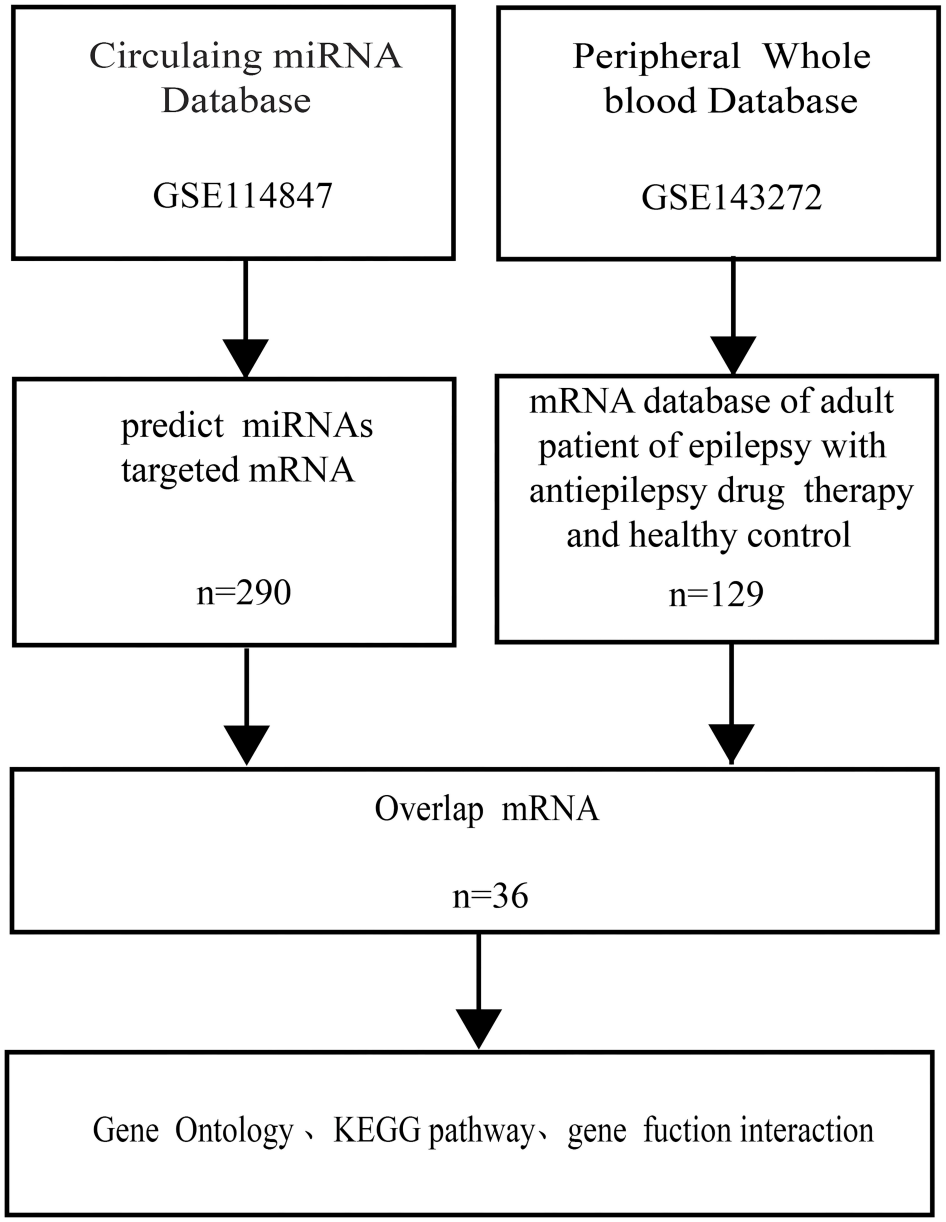
Flowchart of the bioinformation. miRNA indicated microRNA; KEGG indicated Kyoto Encyclopedia of Genes and Genomes.

As part of this study, we combined two genome-wide datasets of adult epilepsy patients, including a plasma miRNA dataset and an entire peripheral whole-blood mRNA dataset, to gain an overall evaluation of the effect that circulating miRNAs have on adult epilepsy patients' peripheral whole-blood mRNAs (Figure 1).

## Subjects and methods

### Subjects

In the current study, the microarray data of the expression of the gene and GSE143272 epileptic datasets were downloaded from NCBI GEO (available online: https://www.ncbi.nlm.nih.gov/geo/) having a total of 89 subjects, i.e., 40 epileptic and 49 healthy controls. All the patients had epilepsy etiology prior to admission and were on drug therapy. Blood samples were taken 24 h following a single seizure, which included both focal and generalized seizures, and were analyzed (Table 1). Healthy individuals falling within the same age range, i.e., 18–50 years, with no seizure history or any other chronic medical ailment, were also enrolled as a control group for the study. Furthermore, the GSE114847 platform was also retrieved having the expression data of miRNA acquired from the blood sample of 32 adult patients (both epilepsy *n* = 32 and healthy control *n* = 32), respectively. All patients had refractory focal epilepsy prior to admission and were on polydrug therapy. In addition, 32 non-fasting male and female healthy control volunteers were recruited for the study. A blood sample was taken from each patient within 24 h of the seizure onset for all of the individuals who had expressed focal seizures (Table 2). Supplementary Tables S1, S2 summarize the diagnosis, demographics, medication, and origin of each sample for all patients and control groups that satisfied the diagnostic criteria for epilepsy. Blood samples were collected in the same period of time between healthy control and patients.

**Table 1:** Descriptive data of the mRNA study groups.

	**Patients** **(*n* = 40)**	**Health control** **(*n* = 49)**	***p*** **-value**
Ages (years)	26.12 ± 6.98	28.16 ± 8.30	0.22
Male/female ratio	25/15	23/26	0.47
Seizure type	Focal/generalized		
Drug therapy	Yes		

**Table 2:** Descriptive data of the miRNA study groups.

	**Patients** **(*n* = 32)**	**Health control** **(*n* = 32)**	***p*** **-value**
Ages (years)	41.28 ± 16.59	36.93 ± 10.10	0.21
Male/female ratio	19/13	18/14	1
Seizure type	Focal		
Drug therapy	Yes		

### Methods

#### Acquiring the adult epilepsy-related mRNAs

The obtained original raw microarray data were further subjected to normalization and gene expression level identification analysis through GEO2R (http://www.ncbi.nlm.nih.gov/geo/geo2r) of the R package. The differential expression of the GEO series may be discovered using an online program that enables the comparison of two or more groups of samples. It automatically calculates the false discovery rate (FDR). Multiple *t*-tests were used to identify genes with statistical significance while also allowing for FDR correction. These genes were characterized as genes with a |log2(Fold Change)| > 1.5 and *p*-value < 0.05. Additionally, to further comprehend how these genes interact with one another and visualized metabolic pathways, they were utilized to map all of the genes with changed expression in adult epilepsy mRNA.

#### Gene Ontology (GO) and Kyoto Encyclopedia of Genes and Genomes (KEGG) pathway analysis

To analyze the identified mRNAs in terms of DEGs at potential molecular functions, cellular components and biological processes associated with epilepsy and control samples were elucidated through Gene Ontology (GO) enrichment analysis. In bioinformatics, GO analysis is a standard approach for gene annotation and transcriptome data analysis. Furthermore, the Kyoto Encyclopedia of Genes and Genomes (KEGG) metabolic enrichment pathway analysis was performed to explore the potential pathways involved in an epileptic seizure. KEGG is a database that helps researchers better understand the biological system's sophisticated functions and utilities. Eventually, the Database for Annotation, Visualization, and Integrated Discovery (DAVID, http://david.abcc.ncifcrf.gov/) was used to analyze the GO and KEGG pathway databases for DEGs.

#### Prediction of miRNA transcription factor

Transcription factors (TFs) are DNA-binding proteins that exert indispensable influence in regulating the expression of the gene and cancer-associated pathways (15). Mounting evidence suggests that some TFs interact with miRNAs to impact the transcriptome of target genes (16). In this study, TFs that target the seven most upregulated miRNAs and three most downregulated miRNAs, as ranked by *p*-value, were predicted by TransmiR v.2.0 database store regulatory relations between human TFs and miRNAs.

## Results

### Differently expressed circulating miRNAs and peripheral whole-blood mRNAs

According to the findings of the previous study, a group of circulating miRNAs that constitute possible molecular biomarkers of focal seizure have been identified (17). By comparing the findings of plasma samples taken after epilepsy with plasma samples collected from controls, we were able to identify miRNAs with differential expression. When compared with control samples, patient samples showed a fold change of >1.5 or −1.5 in the expression of 10 miRNAs, with 7 miRNAs having an upregulation and 3 miRNAs having a downregulation (Table 3).

**Table 3:** Differently expressed circulating AD-miRNA of patients of focal seizure.

	**miRNA**
Up-regulated miRNA	has-miR-654-3p, hsa-miR-543
	has-miR-144-3p, hsa-miR-323a-3p
	has-miR-323b-3p, hsa-miR-328-3p
	hsa-miR-122-5p
Down-regulated miRNA	hsa-miR-342-5p, hsa-miR-150-5p
	hsa-miR-339-5p

**Figure 2:**
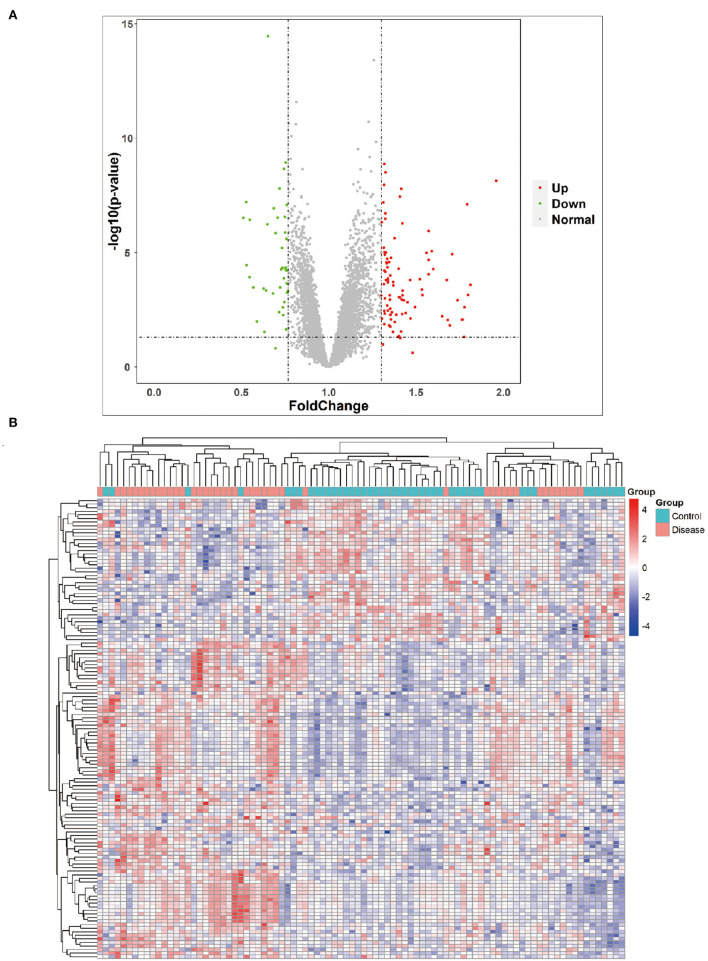
Volcano plot and heat map. **(A)** Volcano plot showing the most significant genes found by univariate analysis. The 40 downregulated genes and upregulated genes were significantly different in the adult patients of epilepsy group compared to the adult healthy group. **(B)** Collected RNA-seq data analysis and heat map from adult epilepsy patients sample and control.

Furthermore, the 89 DEGs collected from epilepsy and healthy control samples were then used in hierarchical cluster analysis. It was observed that among these 89 DEGs, 40 were disease-associated DEGs and 49 were control-associated DEGs, respectively. However, seizure-related adult patients had 79 upregulated and 40 downregulated genes (Figure 2A), and the total number of reads collected from each sample is indicated in the Figure 2B. Supplementary Tables S3–S5 include the unprocessed data.

### Detection of AD-mRNA and AD-miRNA of patients with epileptic seizures

**Figure 3:**
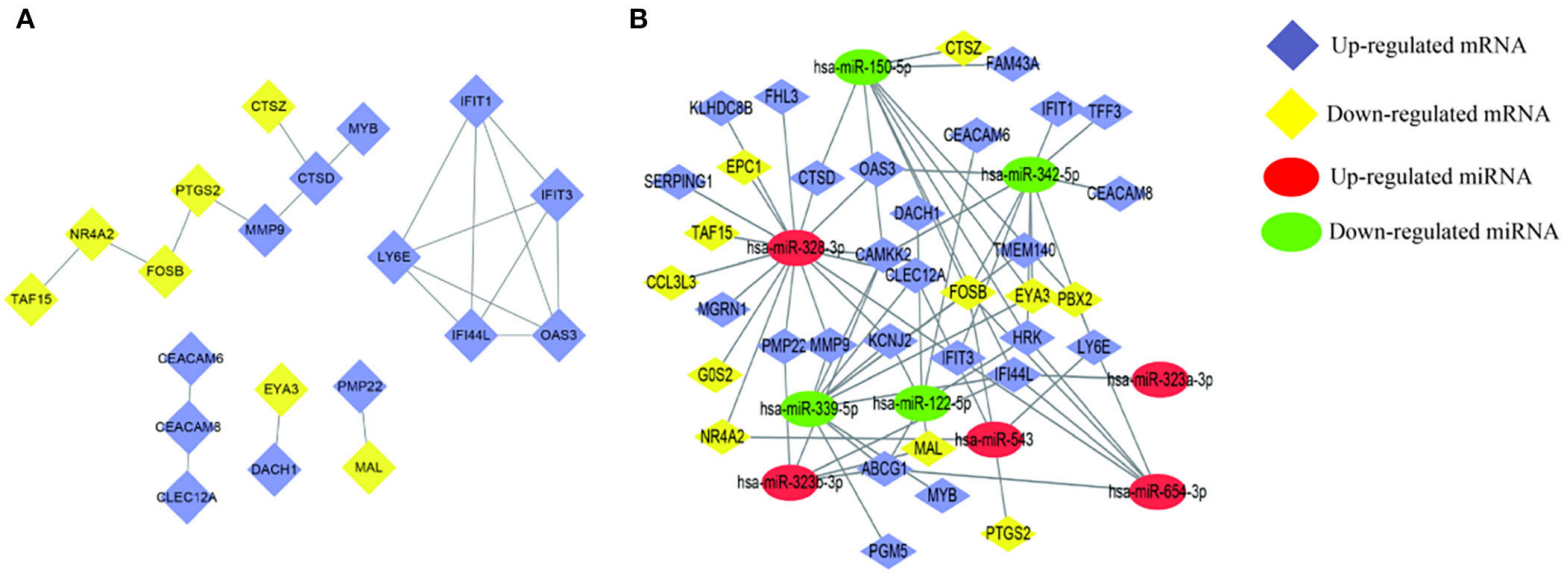
Detection of AD-mRNA and AD-miRNA of patients of epileptic seizures. **(A)** Interactive relationships of AD-mRNAs, including five modules 20 nodes and 21 edges. **(B)** Networking of the regulatory relationships between AD-miRNAs and AD-mRNAs. Lines indicate the regulatory from one miRNA to the targets. “has-” indicates Homo sapiens.

The circulating AD-miRNAs have their target mRNAs and were identified by the MirWalk tool. Consequently, 36 target mRNAs were identified in peripheral whole blood that had been overlapped after intersecting with the peripheral whole-blood mRNAs (Table 4). To display the interactions among the 36 overlapping AD-mRNAs, the STRING program was employed. It resulted in the protein-protein interaction (PPI) network having 36 nodes and 21 edges (Figure 3A). Module 1 was made up of eight nodes and seven edges consisting of specific Alzheimer's disease-associated mRNAs, such as the matrix metalloproteinase-9 (MMP9), the protein fosB (FOSB), the interferon-induced protein with tetratricopeptide repeats 1 (ITFIT1), transcriptional activator Myb (MYB), the cathepsin D (CTSD), the nuclear receptor subfamily 4 group A member 2 (NR4A2), TATA-binding protein-associated factor 2N (TAF15), and the prostaglandin G/H synthase 2 (PTGS2). Whereas, the second module was observed to have five nodes and 10 edges mainly enriched in immune-related pathways, such as 2'-5'-oligoadenylate synthase 3 (OAS3), lymphocyte antigen 6E (LY6E), interferon-induced protein 44-like (ITIF4), interferon-induced protein with tetratricopeptide repeats 3 (IFIT3), lymphocyte antigen 6E (IFI44L), and tetratricopeptide repeats 1 (ITIF1). However, the third module consisted of three nodes and two edges, having proteins such as carcinoembryonic antigen-related cell adhesion molecule family (CEACAM6 CEACAM8) and C-type lectin domain family 12 member A (CLEC12A). Additionally, Modules 4 and 5 were found to consist of two nodes and one edge, respectively, such as Myelin and lymphocyte protein (MAL), Eyes absent homolog 3 (EYA3), Dachshund homolog 1 (DACH1), and Peripheral myelin protein 22 (PMP22). According to these findings, AD-miRNAs found in the peripheral blood of epileptic seizure patients may play an important role in pathophysiological processes. As part of our research, we examined the correlation that exists between AD-miRNA and AD-mRNA (Table 5). It was discovered that AD-mRNAs and AD-miRNAs had a biological mechanism in parallel (Figure 3B).

**Table 4:** AD-miRNAs and AD-mRNAs of patients of epileptic seizures.

	**AD-miRNA**	**AD-mRNA**
Up regulated miRNA	has-miR-654-3p	ABCG1, IFIT3, HRK, PBX2, FOSB
	has-miR-543	LY6E, CLEC12A, PTGS2, NR4A2
		FOSB
	has-miR-323a-3p	IFI44L
	has-miR-323b-3p	CAMKK2, ABCG1, IFI44L, PMP22
		MAL
	has-miR-328-3p	CTSD, MGRN1, FHL3, CLEC12A
		CAMKK2, KCNJ2, KLHDC8B, IFIT3
		SERPING1, OAS3, MMP9, PMP22
		EPC1, TAF15, NR4A2, CCL3L3
	has-miR-122-5p	ABCG1, CEACAM6, PACH1, KCNJ2
		HRK, MAL
Down regulated miRNA	has-miR-342-5p	LY6E, CAMKK2, IFIT3, IFIT1, OAS3
		HRK, TFF3, CEACAM8, EYA3, FOS8
		MAL
	has-miR-150-5p	CTSD, CAMKK2, FAM43A, IFI44L
		HRK, CTS2, PBX2, EYA3, FOSB
	has-miR-339-5p	CLEC12A, CAMKK2, TMEM140
		ABCG1, KCNJ2, MYB, IFI144L, MAL
		IFIT1, PGM5, MMP9, EYA3, FOSB

**Table 5:** Thirty-six overlapping AD-mRNAs.

**Gene symbol**	**Description**	**Up/down gene**
ABCG1	ATP binding cassette subfamily G member 1	Up
IFI44L	Interferon induced protein 44 like	Up
IFIT3	Interferon induced protein with tetratricopeptide repeats 3	Up
HRK	harakiri BCL2 interacting protein	Up
PBX2	PBX homeobox 2	Up
LY6E	Lymphocyte antigen 6 family member E	Up
CLEC12A	C-type lectin domain family 12 member A	Up
PMP22	Peripheral myelin protein 22	Up
MAL	mal T cell differentiation protein	Up
CAMKK2	calcium/calmodulin dependent protein kinase kinase 2	Up
CTSD	Cathepsin D	Up
MGRN1	Mahogunin ring finger 1	Up
FHL3	Four a half LIM domains 3	Up
KLHDC8B	Kelch domain containing 8B	Up
SERPING1	Serpin family G member 1	Up
KCNJ2	Potassium inwardly rectifying channel subfamily J member 2	Up
OAS3	2′-5′-oligoadenylate synthetase 3	Up
MMP9	Matrix metallopeptidase 9	Up
DACH1	Dachshund family transcription factor 1	Up
IFIT1	Interferon induced protein with tetratricopeptide repeats 1	Up
TFF3	Trefoil factor 3	Up
CEACAM8	CEA cell adhesion molecule 8	Up
FAM43A	Family with sequence similarity 43 member A	Up
TMEM140	Transmembrane protein 140	Up
MYB	MYB proto-oncogene transcription factor	Up
PGM5	Phosphoglucomutase 5	Up
FOSB	FosB proto-oncogene AP-1 transcription factor subunit	Down
PTGS2	Prostaglandin-endoperoxide synthase 2	Down
NR4A2	Nuclear receptor subfamily 4 group A member 2	Down
EPC1	Enhancer of polycomb homolog 1	Down
TAF15	TATA-box binding protein associated factor 15	Down
G0S2	G0/G1 switch 2	Down
CCL3L3	C-C motif chemokine ligand 3 like 3	Down
CEACAM6	CEA cell adhesion molecule 6	Down
EYA3	EYA transcriptional coactivator phosphatase 3	Down
CTSZ	Cathepsin Z	Down

### Functional enrichment analysis of the AD-mRNAs of patients with epileptic seizures

Database for annotation, visualization, and integrated discovery was used to conduct GO and KEGG pathway enrichment analyses on 36 overlapping AD-mRNAs to learn more about their function. Importantly, biological process, molecular function, and cell components are one of the most common GO categories.

### GO analysis and KEGG pathway enrichment analysis of the AD-mRNAs of patients with epileptic seizures

**Figure 4:**
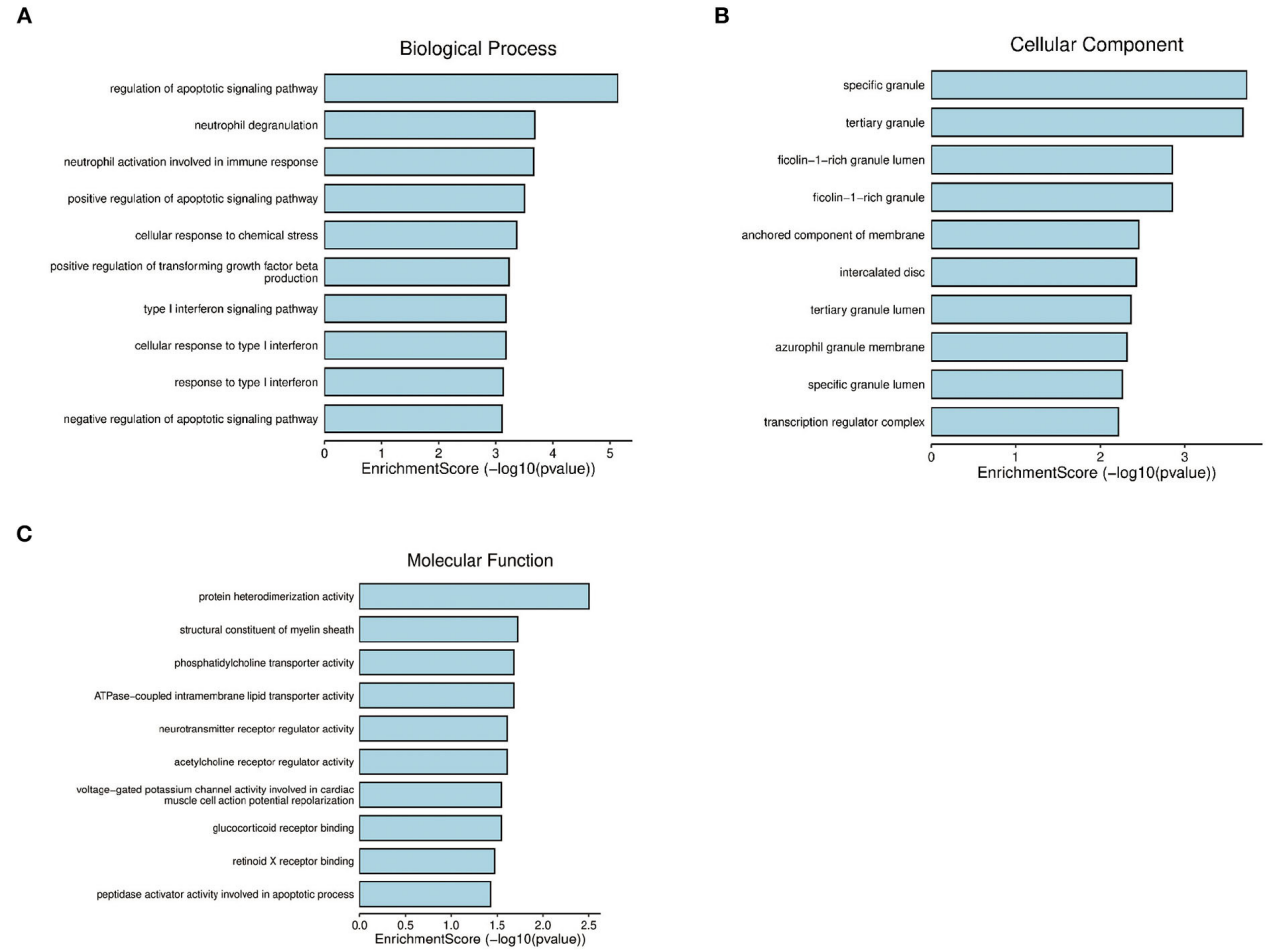
Gene ontology analysis for AD-mRNA. **(A)** The enrichment of genes in the 10 most significant terms of biological process. **(B)** The enrichment of genes in the 10 most significant terms of cellular component. **(C)** The enrichment of genes in the 10 most significant terms of molecular function.

**Figure 5:**
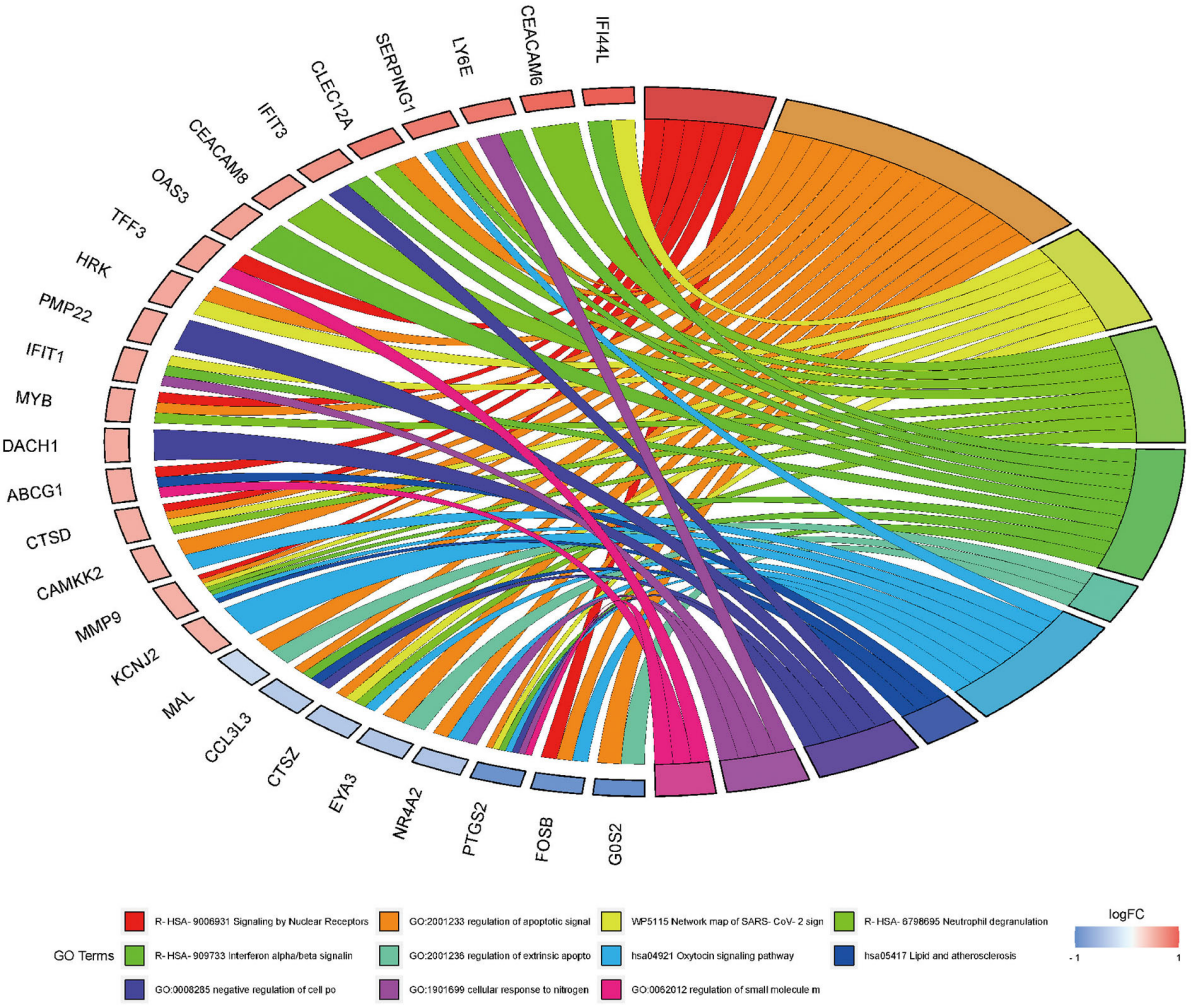
Gene ontology analysis for AD-mRNA GO Chord plot of top 10 ranked overrepresented GO terms belonging to the biological process. The genes are linked to their assigned terms *via* colored ribbons. Genes are ordered according to the observed log-fold change (logFC), which is displayed in descending intensity of red squares displayed next to the selected genes.

Next, to obtain a more comprehensive understanding and to identify the functional categories of the DEGs, the data were clustered through GO analysis in DAVID. The top 10 significantly enriched GO terms with a *p*-value < 0.05 that were divided into the biological process (BP), cellular component (CC), and molecular function (MF) ontologies are illustrated in Figures 4A–C. Regarding the BP ontology, the highly enriched GO terms of AD-DEGs were mainly related to the regulation of apoptotic signaling pathways, neutrophil degranulation, and type I interferon signaling pathways. In the CC ontology, we found that the majority of overlapping AD-mRNAs were associated with transcription regulator complex, specific granule, and tertiary granule. In the MF ontology of overlapping AD-mRNAs, the protein binding items constituted most of the enriched GO categories, including protein heterodimerization activity. The details are shown in Supplementary Table S6. Pathways that included regulation of apoptotic signal and interferon alpha/beta signaling were significantly associated with these target genes (Figure 5). Highly enriched GO terms of overlapping up-ADmRNAs were mainly related to defense response to the virus (*p* < 0.05). The majority of overlapping down-AD-mRNAs were associated with positive regulation of neuron apoptotic process and negative regulation of protein binding (*p* < 0.05). The details are shown in Table 6. These roles were critical to the control of the expression of AD-mRNA in the pathogenesis of adult epilepsy.

**Table 6:** Gene ontology analysis for overlapping-AD-mRNA.

	**Category**	**Term**	**Count**	**P-value**	**Genes**
Up overlapping-AD MRNAs	GOTERM_BP_DIRECT	GO:0051607~defense response to virus	4	0.0022173	OAS3, IFIT1, IFI44L, IFIT3
	GOTERM_BP_DIRECT	GO:0009615~response to virus	3	0.007055231	OAS3, IFIT1, IFIT3
	GOTERM_BP_DIRECT	GO:0090200~positive regulation of release of cytochrome c from mitochondria	2	0.03152324	HRK, MMP9
	GOTERM_BP_DIRECT	GO:0034614~cellular response to reactive oxygen species	2	0.041452648	MMP9, CAMKK2
	GOTERM_BP_DIRECT	GO:0045071~negative regulation of viral genome replication	2	0.051284836	OAS3, IFIT1
	GOTERM_BP_DIRECT	GO:0043065~positive regulation of apoptotic process	3	0.053413346	HRK, CTSD, MMP9
	GOTERM_CC_DIRECT	GO:0014704~intercalated disc	2	0.053653465	PGM5, KCNJ2
	GOTERM_CC_DIRECT	GO:1904724~tertiary granule lumen	2	0.060030814	CTSD, MMP9
	GOTERM_CC_DIRECT	GO:0005615~extracellular space	6	0.060036453	OAS3, TFF3, SERPING1, CEACAM8, CTSD, MMP9
	GOTERM_CC_DIRECT	GO:0001725~stress fiber	2	0.078917151	FHL3, PGM5
	GOTERM_CC_DIRECT	GO:0070821~tertiary granule membrane	2	0.078917151	CLEC12A, CEACAM8
	GOTERM_CC_DIRECT	GO:0035579~specific granule membrane	2	0.097440195	CLEC12A, CEACAM8
	GOTERM_MF_DIRECT	GO:0005515~protein binding	21	0.026354819	HRK, CLEC12A, MGRN1, PBX2, FHL3, IFIT1, TMEM140, MMP9, IFIT3, DACH1, OAS3, MYB, PMP22, TFF3, SERPING1, FAM43A, CEACAM8, CTSD, KCNJ2, ABCG1, LY6E
Down overlapping-AD MRNAs	GOTERM_BP_DIRECT	GO:0043525~positive regulation of neuron apoptotic process	2	0.022681888	CTSZ, MYB
	GOTERM_BP_DIRECT	GO:0032091~negative regulation of protein binding	2	0.026951866	CTSZ, IFIT1
	GOTERM_CC_DIRECT	GO:0005667~transcription factor complex	2	0.086798207	DACH1, EYA3

**Figure 6:**
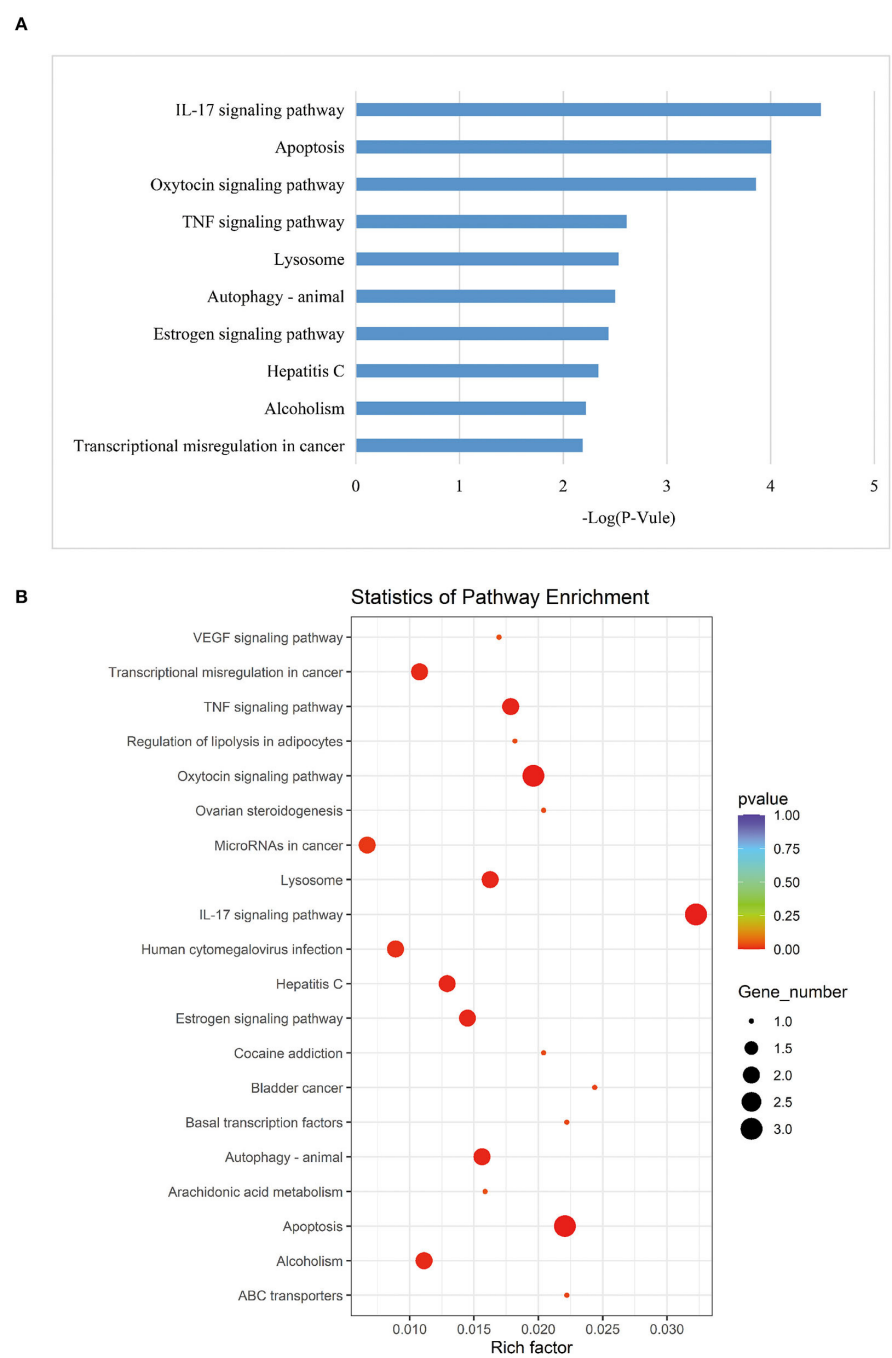
KEGG analysis. **(A)** Top 10 significant enriched pathway, the length of each bar chart indicates the enrichment for corresponding KEGG pathway. **(B)** The most 20 KEGG pathways were presented. The y-axis and x-axis indicate pathway name and rich factor, respectively. The size of circle dot means gene number.

**Figure 7:**
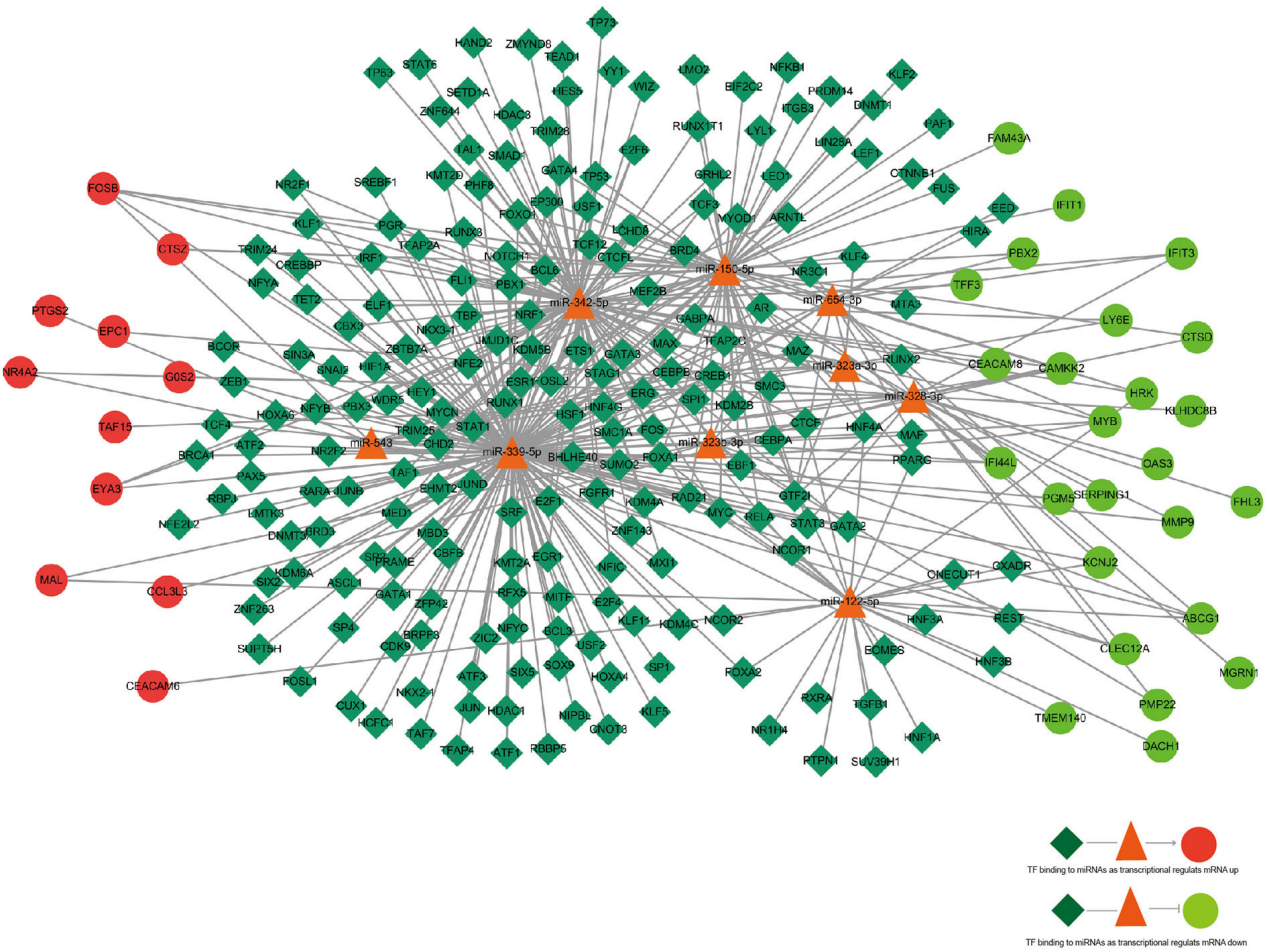
The regulatory network of TF–miRNA–mRNA. TF, miRNA, and mRNA were indicated to diamond, triangle, and circle, respectively. The color of red represents high expression and green represents low expression. Arrows indicated miRNAs regulating mRNA, and straight line indicated miRNAs deregulating mRNA. The azure color line indicated TF binding to miRNA.

According to the KEGG pathways enrichment analysis, the top 20 enriched pathways were displayed most prominently as highlighted in Table 4 and Figure 3B. It was observed that the apoptosis pathway, estrogen signaling pathway, TNF signaling pathway, alcoholism, Hepatitis C, oxytocin signaling pathway, IL-17 signaling pathway, transcriptional misregulation in cancer, autophagy—animal, and lysosome were among the top 10 significant pathways associated with the overlapping AD-mRNAs. The fold enrichment of AD-mRNAs involvement in each pathway was shown by a bar chart (Figures 6A,B, Supplementary Table S7). Subsequently of the annotations of DEGs, it is possible to do additional research into specific processes and pathways that were implicated in the occurrence of epileptic seizures. Prediction results from TransmiR for the significant overlapping DE-miRNAs are shown in Figure 7 and Supplementary Table S8. It can be observed that several TFs, such as GTF2I, STAT6, NCOR2, KLF2, LIN28A, WIZ, ZNF644, and HNF4A, could target up to four miRNAs (*p* < 0.05; Supplementary Table S9).

## Discussion

Considering the etiology of epileptic seizures is intricate and relies on several variables, in this work, we primarily studied the regulatory influence of circulating miRNAs on the mRNAs in peripheral whole blood. Consequently, an all-encompassing depiction of the physiopathology response to epileptic convulsions may be constructed. Although the majority of miRNAs are found inside cells, circulating miRNAs have been shown to have an essential role in the progression of disease (18). Furthermore, investigations have demonstrated that circulating miRNAs may affect the expression of a gene by influencing intracellular mRNA transcription (19). Circulating miRNAs may influence mRNA transcription of cells in peripheral whole blood, the cerebrovascular endothelium, and brain tissue in epileptic convulsions. In this research, we look at the GSE114847 and GSE143772 profile datasets. Following that, KEGG pathway and GO enrichment studies were carried out. Defense response to the virus was all associated with overlapping upregulated AD-mRNAss, according to the GO analysis, while overlapping downregulated AD-mRNAs were associated with positive regulation of neuron apoptotic process. The findings suggest the existence of a common relationship between epilepsy and viral infection. Following the results of the KEGG analysis, the AD-DEGs were predominantly enriched in the alcoholism, TNF signaling pathway, apoptosis, estrogen signaling pathway, oxytocin signaling pathway, autophagy-animal, hepatitis C, lysosome, IL-17 signaling pathway, and transcriptional misregulation in cancer pathways. According to the pathway analysis results, infection of the nervous system mainly relates to the reasons for epileptic cases in the analysis.

In chronic infection, type I interferons (IFN-Is) are emerging as major drivers of inflammation and immunosuppression. Multiple patterns or damage recognition receptors generate IFN-Is in viral infection, frequently at high levels (20). Epilepsy may arise as a result of CNS infections that occur in the adult years. Infections of the central nervous system (CNS) are a primary cause of epileptic seizures. Furthermore, seizures may be the primary sign of an infestation (21). Neuronal hyperexcitability, neuronal loss, and gliosis are just a few of the changes that can occur during the latent phase between infection and the onset of seizures. These changes, as well as other brain alterations, can lead to spontaneous recurrent seizures (22, 23) and therefore, epileptic seizures should encompass all of these pathways.

According to the findings of this research, three upregulated mRNAs were found to be associated with the IFN-Is signaling pathway and have the ability to stimulate the biological process of chronic infection in epileptic seizures. Seizures have previously been linked to high doses of interferon-gamma/gamma receptor (IFN α/β receptor knockout mice; AG129 strain) causing progressive hind limb paralysis and severe seizure-like activity during the acute phase of disease (22, 23), but there have also been reports of grand mal seizures occurring during therapy with low doses of IFN-α. However, there have been only a few such investigations on the relationship between Type I interferons and seizures. Our findings demonstrate that IFIT3, IFIT1, and OAS3, and STRING analysis shows that the gene network of IFIT3, IFIT1, IFI44L, OAS3, and LY6E are implicated in seizures during adult epilepsy, which suggests that they may impact the therapeutic efficacy of antiepileptic medicines and may be possible biomarkers for antiepileptic treatment. A previous study on ghrelin, which is an orexigenic peptide synthesized by endocrine cells of the gastric mucosa, has hinted that it plays a major role in inhibiting seizures. However, up to now, there has been no general consensus regarding the differences in interictal ghrelin levels between adult epilepsy patients and healthy subjects (24).

We observed that adult patients with epileptic seizures had peripheral whole blood that included miR-654-3p, miR-323a-3p, miR-323b, miR-3283p, miR-342-5p, miR-150-5p, miR-3395p and IFIT3, IFIT1, IFI44L, OAS3, and LY6E in the pathways connected to viral infection. As a result, we hypothesized that the body's susceptibility to infection by a virus increased during seizures. During one-set seizures, the circulating miRNA regulated the target mRNA's post-infection response. The early different expression of miRNA may represent a directed response to infection that upregulates vital pathways such as the Type 1 signal pathway and the response to the virus.

The significance of the inflammatory response has been explored in recent research, and pro-inflammatory cytokines have long been suspected of playing a significant role in the pathophysiology and epileptogenicity. Human and experimental seizures have been demonstrated to enhance the expression of IL-1R1 and TLR4 in neurons, astrocytes, and the BBB endothelium, which provides evidence that these receptors are upregulated (25–29). Some of the cytokines that have been found in the sera of persons with seizures include interleukin (IL)-1, tumor necrosis factor (TNF)-α, and IL-6 (30, 31). Indeed, according to previous research, epilepsy sufferers' plasma miRNAs were shown to target a wide range of pathways including growth factor signaling, apoptosis-associated signaling, and the oxytocin signaling pathway. On the other hand, an integrated analysis revealed that the IL-17 signaling pathway merited further study. Interleukin 17, often known as IL-17, is the newest subclass of cytokines and is the most common cytokine in the body. An interleukin 17-induced release of specific chemokines (e.g., IL-1α, IL-6, and TNF) and its unique receptor-ligand system makes it one of the most potent cytokines in the body (32). In diseases such as multiple sclerosis and ischemic brain damage, IL-17/IL-17 receptor (IL-17R) interaction was shown to induce neuronal injury by bridging the gap between innate and adaptive immunity *in vivo* (33–35). Only two studies found a link between the IL-17 system and epilepsy-related cortical tubers of tuberous sclerosis complex (TSC) and focal cortical dysplasia (FCDs) (36, 37). As a result, the IL-17 signaling pathway may have aided in the progression of epilepsy. SERPING1 was the most substantially differently expressed mRNA (log FC = 0.65, *p* = 8.83E-05) among 36 overlapped mRNA. Because SERPING1 is strongly expressed during seizures, it may be a vulnerable epilepsy prediction factor. According to Maja Kapczynaska et al., complement dysregulation was found in a regularly collected clinical cohort of epilepsy patients (38). SERPING1 is a C1 esterase inhibitor (C1-INH) that regulates C3 activation by blocking the C1r/C1s proteases of the classical and lectin pathways. It is Food and Drug Administration (FDA) authorized to treat hereditary angioedema in humans (39, 40). Early therapy with C1-INH improves weight gain in epilepsy animal models. As a result, microgliosis was increased by acute C1-INH therapy 3 days after SE, and synaptic proteins were depleted from the hippocampus 14 days after SE. We found that in the peripheral whole blood of adult epilepsy patients, SERPING1 was downregulated, whereas miR-328-3p, a circulating microRNA, was upregulated. The control of circulating miRNA by SERPING1 may thus play an important role in epilepsy. The involvement of SERPING1 and its related regulatory miRNAs in epileptic seizures needs additional study. TFs have crucial actions on the transcription regulation of genes *via* binding the promoters and distal cis-regulatory elements. A great number of TFs were reaped from the prediction of TransmiR. Particularly, we hypothesized that these TFs might be engaged in seizures-induced expression change of the most significant miRNAs at the transcription level. GTF2I was predicted by TransmiR to target five of the seven upregulated miRNAs, which hinted at the positive regulatory relationship between GTF2I and the five upregulated miRNAs.

However, there are several limits to our research. Although the datasets used in this investigation were gathered from patients, there are constraints to conducting trials to prove the relationships. Animal models will be used in future investigations to confirm the preservation of these miRNA–mRNA pairings along with their functional connections. Second, circulating miRNAs may reside in additional possible target tissues or organs, such as the epileptic brain tissue and cerebrovascular endothelium in addition to peripheral whole blood, necessitating more investigation.

## Data availability statement

Publicly available datasets were analyzed in this study. This data can be found at: National Center for Biotechnology Information (NCBI) Gene Expression Omnibus (GEO), https://www.ncbi.nlm.nih.gov/geo/, GSE143272, and GSE114847.

## Ethics statement

Ethical review and approval was not required for the study on human participants in accordance with the local legislation and institutional requirements. Written informed consent from the patients/participants or patients/participants' legal guardian/next of kin was not required to participate in this study in accordance with the national legislation and the institutional requirements.

## Author contributions

QW, CW, and JW contributed to conception and design of the study. FX organized the database. YM performed the statistical analysis. JW wrote the first draft of the manuscript. JW and QW wrote sections of the manuscript. All authors contributed to manuscript revision, read, and approved the submitted version.

## Funding

This study was supported with grants from the Science and Technology Development Foundation of Nanjing Medical University (NMUB2020250).

## Conflict of interest

The authors declare that the research was conducted in the absence of any commercial or financial relationships that could be construed as a potential conflict of interest.

## Publisher's note

All claims expressed in this article are solely those of the authors and do not necessarily represent those of their affiliated organizations, or those of the publisher, the editors and the reviewers. Any product that may be evaluated in this article, or claim that may be made by its manufacturer, is not guaranteed or endorsed by the publisher.
