# Supplementary material for: The viral and inflammation hypothesis of epileptic seizures based on bioinformatic study of circulating miRNAs and peripheral whole-blood mRNAs of adult epilepsy patients
Source: Front Neurol. 2022 Sep 12;13:909142. doi: 10.3389/fneur.2022.909142 (PMC9510610; doi:10.3389/fneur.2022.909142)
Supplement: Supplementary file 5 [file Table_5.docx]

| ID | Description | GO term | Regulation |
| --- | --- | --- | --- |
| ZDHHC12 | zinc finger DHHC-type palmitoyltransferase 12 | GO:0097116 gephyrin clustering involved in postsynaptic density assembly;GO:0097107 postsynaptic density assembly;  GO:0098698 postsynaptic specialization assembly | Up |
| CTSD | cathepsin D | GO:0042159 lipoprotein catabolic process;GO:0019886 antigen processing and presentation of exogenous peptide antigen via MHC class II;GO:0002495 antigen processing and presentation of peptide antigen via MHC class II | Up |
| HES4 | hes family bHLH transcription factor 4 | GO:0009952 anterior/posterior pattern specification;GO:0003002 regionalization;GO:0007389 pattern specification process | Up |
| BSG | basigin (Ok blood group) | GO:0046689 response to mercury ion;GO:0070593 dendrite self-avoidance;GO:0046697 decidualization | Up |
| TYMP | thymidine phosphorylase | GO:0046074 dTMP catabolic process;GO:1905333 regulation of gastric motility;GO:0046073 dTMP metabolic process | Up |
| PPDPF | pancreatic progenitor cell differentiation and proliferation factor | GO:0030154 cell differentiation;GO:0048869 cellular developmental process;GO:0032502 developmental process | Up |
| LSP1 | lymphocyte specific protein 1 | GO:0098760 response to interleukin-7;GO:0098761 cellular response to interleukin-7;GO:0006968 cellular defense response | Up |
| HLA-C | major histocompatibility complex, class I, C | GO:0002484 antigen processing and presentation of endogenous peptide antigen via MHC class I via ER pathway;GO:0002486 antigen processing and presentation of endogenous peptide antigen via MHC class I via ER pathway, TAP-independent;GO:0019885 antigen processing and presentation of endogenous peptide antigen via MHC class I | Up |
| LGALS9 | galectin 9 | GO:0070239 regulation of activated T cell autonomous cell death;GO:0070241 positive regulation of activated T cell autonomous cell death;GO:0032832 regulation of CD4-positive, CD25-positive, alpha-beta regulatory T cell differentiation involved in immune response | Up |
| RNF19B | ring finger protein 19B | GO:0042267 natural killer cell mediated cytotoxicity;GO:0002228 natural killer cell mediated immunity;GO:0001909 leukocyte mediated cytotoxicity | Up |
| MGRN1 | mahogunin ring finger 1 | GO:0043951 negative regulation of cAMP-mediated signaling;GO:0043949 regulation of cAMP-mediated signaling;GO:0045879 negative regulation of smoothened signaling pathway | Up |
| FHL3 | four and a half LIM domains 3 | GO:0007517 muscle organ development;GO:0061061 muscle structure development;GO:0030036 actin cytoskeleton organization | Up |
| LY6E | lymphocyte antigen 6 family member E | GO:0048242 epinephrine secretion;GO:0050432 catecholamine secretion;GO:0048241 epinephrine transport | Down |
| ZBP1 | Z-DNA binding protein 1 | GO:0060545 positive regulation of necroptotic process;GO:0062100 positive regulation of programmed necrotic cell death;GO:2000659 regulation of interleukin-1-mediated signaling pathway | Down |
| HSPA1B | heat shock protein family A (Hsp70) member 1B | GO:0010286 heat acclimation;GO:0070370 cellular heat acclimation;GO:0070434 positive regulation of nucleotide-binding oligomerization domain containing 2 signaling pathway | Down |
| CLEC12A | C-type lectin domain family 12 member A |  | Down |
| CD24 | CD24 molecule | GO:0032595 B cell receptor transport within lipid bilayer;GO:0032597 B cell receptor transport into membrane raft;GO:0032599 protein transport out of membrane raft | Down |
| CAMKK2 | calcium/calmodulin dependent protein kinase kinase 2 | GO:0061762 CAMKK-AMPK signaling cascade;GO:0099004 calmodulin dependent kinase signaling pathway;GO:1903599 positive regulation of autophagy of mitochondrion | Down |
| KLHDC8B | kelch domain containing 8B | GO:1902410 mitotic cytokinetic process;GO:0032506 cytokinetic process;GO:0000281 mitotic cytokinesis | Down |
| TMEM140 | transmembrane protein 140 |  | Down |
| TCN1 | transcobalamin 1 | GO:0006824 cobalt ion transport;GO:0015889 cobalamin transport;GO:0051180 vitamin transport | Down |
| IFIT2 | interferon induced protein with tetratricopeptide repeats 2 | GO:0035457 cellular response to interferon-alpha;GO:0035455 response to interferon-alpha;GO:0008637 apoptotic mitochondrial changes | Down |
| GAPT | GRB2 binding adaptor protein, transmembrane | GO:0002322 B cell proliferation involved in immune response;GO:0001782 B cell homeostasis;GO:0002381 immunoglobulin production involved in immunoglobulin-mediated immune response | Down |
| TMEM121B | transmembrane protein 121B |  | Down |
| ABCG1 | ATP binding cassette subfamily G member 1 | GO:0071403 cellular response to high density lipoprotein particle stimulus;GO:0034436 glycoprotein transport;GO:0045542 positive regulation of cholesterol biosynthetic process | Down |
| PLSCR1 | phospholipid scramblase 1 | GO:0006659 phosphatidylserine biosynthetic process;GO:2000371 regulation of DNA topoisomerase (ATP-hydrolyzing) activity;GO:2000373 positive regulation of DNA topoisomerase (ATP-hydrolyzing) activity | Down |
| RNASE2 | ribonuclease A family member 2 | GO:0002227 innate immune response in mucosa;GO:0002385 mucosal immune response;GO:0002251 organ or tissue specific immune response | Down |
| HOXB2 | homeobox B2 | GO:0021570 rhombomere 4 development;GO:0021569 rhombomere 3 development;GO:0021546 rhombomere development | Down |
| SAMD9L | sterile alpha motif domain containing 9 like |  | Down |
| SERPING1 | serpin family G member 1 | GO:0001868 regulation of complement activation, lectin pathway;GO:0001869 negative regulation of complement activation, lectin pathway;GO:0045916 negative regulation of complement activation | Down |
| FAM43A | family with sequence similarity 43 member A |  | Down |
| ISG15 | ISG15 ubiquitin like modifier | GO:0032461 positive regulation of protein oligomerization;GO:0032459 regulation of protein oligomerization;GO:0032020 ISG15-protein conjugation | Down |
| CEACAM6 | CEA cell adhesion molecule 6 | GO:1904906 positive regulation of endothelial cell-matrix adhesion via fibronectin;GO:1904904 regulation of endothelial cell-matrix adhesion via fibronectin;GO:0034116 positive regulation of heterotypic cell-cell adhesion | Up |
| MPO | myeloperoxidase | GO:0002148 hypochlorous acid metabolic process;GO:0002149 hypochlorous acid biosynthetic process;GO:1990267 response to transition metal nanoparticle | Up |
| FFAR2 | free fatty acid receptor 2 | GO:0002877 regulation of acute inflammatory response to non-antigenic stimulus;GO:0002879 positive regulation of acute inflammatory response to non-antigenic stimulus;GO:0002752 cell surface pattern recognition receptor signaling pathway | Up |
| DACH1 | dachshund family transcription factor 1 | GO:0060244 negative regulation of cell proliferation involved in contact inhibition;GO:0060242 contact inhibition;GO:0060245 detection of cell density | Up |
| ADGRE1 | adhesion G protein-coupled receptor E1 | GO:0007189 adenylate cyclase-activating G protein-coupled receptor signaling pathway;GO:0007188 adenylate cyclase-modulating G protein-coupled receptor signaling pathway;GO:0002250 adaptive immune response | Up |
| IFIT5 | interferon induced protein with tetratricopeptide repeats 5 | GO:0045071 negative regulation of viral genome replication;GO:0045069 regulation of viral genome replication;GO:0048525 negative regulation of viral process | Up |
| KCNJ2 | potassium inwardly rectifying channel subfamily J member 2 | GO:0014861 regulation of skeletal muscle contraction via regulation of action potential;GO:0090076 relaxation of skeletal muscle;GO:0060075 regulation of resting membrane potential | Up |
| RSAD2 | radical S-adenosyl methionine domain containing 2 | GO:0034165 positive regulation of toll-like receptor 9 signaling pathway;GO:0034157 positive regulation of toll-like receptor 7 signaling pathway;GO:0034155 regulation of toll-like receptor 7 signaling pathway | Up |
| MT2A | metallothionein 2A | GO:0036018 cellular response to erythropoietin;GO:0036015 response to interleukin-3;GO:0036016 cellular response to interleukin-3 | Up |
| SLPI | secretory leukocyte peptidase inhibitor | GO:0035821 modulation of process of another organism;GO:0019731 antibacterial humoral response;GO:0045071 negative regulation of viral genome replication | Up |
| EPSTI1 | epithelial stromal interaction 1 |  | Up |
| ELANE | elastase, neutrophil expressed | GO:0002775 antimicrobial peptide production;GO:0002777 antimicrobial peptide biosynthetic process;GO:0002778 antibacterial peptide production | Up |
| BPI | bactericidal permeability increasing protein | GO:0043031 negative regulation of macrophage activation;GO:0031663 lipopolysaccharide-mediated signaling pathway;GO:0032717 negative regulation of interleukin-8 production | Up |
| MYB | MYB proto-oncogene, transcription factor | GO:0051574 positive regulation of histone H3-K9 methylation;GO:1901533 negative regulation of hematopoietic progenitor cell differentiation;GO:0045624 positive regulation of T-helper cell differentiation | Up |
| IFITM3 | interferon induced transmembrane protein 3 | GO:0032897 negative regulation of viral transcription;GO:0046782 regulation of viral transcription;GO:0035455 response to interferon-alpha | Up |
| ALPL | alkaline phosphatase, biomineralization associated | GO:0034516 response to vitamin B6;GO:0042822 pyridoxal phosphate metabolic process;GO:0071529 cementum mineralization | Up |
| OAS1 | 2'-5'-oligoadenylate synthetase 1 | GO:0071659 negative regulation of IP-10 production;GO:0071658 regulation of IP-10 production;GO:2000342 negative regulation of chemokine (C-X-C motif) ligand 2 production | Up |
| PGLYRP1 | peptidoglycan recognition protein 1 | GO:0032824 negative regulation of natural killer cell differentiation;GO:0032827 negative regulation of natural killer cell differentiation involved in immune response;GO:0032826 regulation of natural killer cell differentiation involved in immune response | Up |
| MCEMP1 | mast cell expressed membrane protein 1 |  | Up |
| HERC5 | HECT and RLD domain containing E3 ubiquitin protein ligase 5 | GO:0032020 ISG15-protein conjugation;GO:0050688 regulation of defense response to virus;GO:0000079 regulation of cyclin-dependent protein serine/threonine kinase activity | Up |
| GBP1 | guanylate binding protein 1 | GO:0010771 negative regulation of cell morphogenesis involved in differentiation;GO:1900025 negative regulation of substrate adhesion-dependent cell spreading;GO:1903077 negative regulation of protein localization to plasma membrane | Up |
| IFI44L | interferon induced protein 44 like | GO:0051607 defense response to virus;GO:0140546 defense response to symbiont;GO:0009615 response to virus | Up |
| CEBPE | CCAAT enhancer binding protein epsilon | GO:0030851 granulocyte differentiation;GO:0030225 macrophage differentiation;GO:0140467 integrated stress response signaling | Up |
| IFIT3 | interferon induced protein with tetratricopeptide repeats 3 | GO:0035457 cellular response to interferon-alpha;GO:0035455 response to interferon-alpha;GO:0051607 defense response to virus | Up |
| IFI6 | interferon alpha inducible protein 6 | GO:0051902 negative regulation of mitochondrial depolarization;GO:1904180 negative regulation of membrane depolarization;GO:0051900 regulation of mitochondrial depolarization | Up |
| ARG1 | arginase 1 | GO:0070953 regulation of neutrophil mediated killing of fungus;GO:0070965 positive regulation of neutrophil mediated killing of fungus;GO:1905541 regulation of L-arginine import across plasma membrane | Up |
| CTSG | cathepsin G | GO:0098784 biofilm matrix organization;GO:0098786 biofilm matrix disassembly;GO:0070946 neutrophil-mediated killing of gram-positive bacterium | Up |
| IFIT1 | interferon induced protein with tetratricopeptide repeats 1 | GO:0019060 intracellular transport of viral protein in host cell;GO:0030581 symbiont intracellular protein transport in host;GO:0051097 negative regulation of helicase activity | Up |
| HP | haptoglobin | GO:2000296 negative regulation of hydrogen peroxide catabolic process;GO:2000295 regulation of hydrogen peroxide catabolic process;GO:0010727 negative regulation of hydrogen peroxide metabolic process | Up |
| DSC2 | desmocollin 2 | GO:0086073 bundle of His cell-Purkinje myocyte adhesion involved in cell communication;GO:0086042 cardiac muscle cell-cardiac muscle cell adhesion;GO:0098911 regulation of ventricular cardiac muscle cell action potential | Up |
| PTGDR2 | prostaglandin D2 receptor 2 | GO:1905937 negative regulation of germ cell proliferation;GO:2000255 negative regulation of male germ cell proliferation;GO:1905936 regulation of germ cell proliferation | Up |
| TMEM176A | transmembrane protein 176A | GO:2001199 negative regulation of dendritic cell differentiation;GO:2001198 regulation of dendritic cell differentiation;GO:1902106 negative regulation of leukocyte differentiation | Up |
| MS4A3 | membrane spanning 4-domains A3 | GO:0051726 regulation of cell cycle;GO:0007166 cell surface receptor signaling pathway;GO:0007165 signal transduction | Up |
| CAMP | cathelicidin antimicrobial peptide | GO:0071224 cellular response to peptidoglycan;GO:0032494 response to peptidoglycan;GO:0042119 neutrophil activation | Up |
| OAS3 | 2'-5'-oligoadenylate synthetase 3 | GO:0035394 regulation of chemokine (C-X-C motif) ligand 9 production;GO:0035395 negative regulation of chemokine (C-X-C motif) ligand 9 production;GO:0071659 negative regulation of IP-10 production | Up |
| HRK | harakiri, BCL2 interacting protein | GO:0090200 positive regulation of release of cytochrome c from mitochondria;GO:0090199 regulation of release of cytochrome c from mitochondria;GO:0010822 positive regulation of mitochondrion organization | Up |
| DEFA4 | defensin alpha 4 | GO:0019732 antifungal humoral response;GO:0051673 membrane disruption in another organism;GO:0035821 modulation of process of another organism | Up |
| PGM5 | phosphoglucomutase 5 | GO:0030239 myofibril assembly;GO:0055002 striated muscle cell development;GO:0006006 glucose metabolic process | Up |
| IRX3 | iroquois homeobox 3 | GO:0003165 Purkinje myocyte development;GO:0003167 atrioventricular bundle cell differentiation;GO:0060932 His-Purkinje system cell differentiation | Up |
| PI3 | peptidase inhibitor 3 | GO:0007620 copulation;GO:0007617 mating behavior;GO:0018149 peptide cross-linking | Up |
| DEFA1B | defensin alpha 1B | GO:0052183 modification by host of symbiont structure;GO:0052187 modification by host of symbiont cellular component;GO:0052337 modification by host of symbiont membrane | Up |
| DEFA3 | defensin alpha 3 | GO:0051673 membrane disruption in another organism;GO:0030520 intracellular estrogen receptor signaling pathway;GO:0035821 modulation of process of another organism | Up |
| DEFA1 | defensin alpha 1 | GO:0052183 modification by host of symbiont structure;GO:0052187 modification by host of symbiont cellular component;GO:0052337 modification by host of symbiont membrane | Up |
| TFF3 | trefoil factor 3 | GO:0030277 maintenance of gastrointestinal epithelium;GO:0010669 epithelial structure maintenance;GO:0022600 digestive system process | Up |
| MMP9 | matrix metallopeptidase 9 | GO:2000697 negative regulation of epithelial cell differentiation involved in kidney development;GO:2001268 negative regulation of cysteine-type endopeptidase activity involved in apoptotic signaling pathway;GO:1900122 positive regulation of receptor binding | Up |
| IFI44 | interferon induced protein 44 | GO:0009615 response to virus;GO:0009617 response to bacterium;GO:0051707 response to other organism | Up |
| DEFA1B | defensin alpha 1B | GO:0052183 modification by host of symbiont structure;GO:0052187 modification by host of symbiont cellular component;GO:0052337 modification by host of symbiont membrane | Up |
| PMP22 | peripheral myelin protein 22 | GO:0032060 bleb assembly;GO:0032288 myelin assembly;GO:0007422 peripheral nervous system development | Up |
| CEACAM8 | CEA cell adhesion molecule 8 | GO:0007157 heterophilic cell-cell adhesion via plasma membrane cell adhesion molecules;GO:0098742 cell-cell adhesion via plasma-membrane adhesion molecules;GO:0098609 cell-cell adhesion | Up |
| OLIG2 | oligodendrocyte transcription factor 2 | GO:0021530 spinal cord oligodendrocyte cell fate specification;GO:0021778 oligodendrocyte cell fate specification;GO:0021779 oligodendrocyte cell fate commitment | Up |
| RNASE3 | ribonuclease A family member 3 | GO:0043152 induction of bacterial agglutination;GO:0002227 innate immune response in mucosa;GO:0002385 mucosal immune response | Up |
| SIGLEC14 | sialic acid binding Ig like lectin 14 | GO:0007155 cell adhesion;GO:0009987 cellular process;GO:0008150 biological_process | Up |
| CCL23 | C-C motif chemokine ligand 23 | GO:2001263 regulation of C-C chemokine binding;GO:2001264 negative regulation of C-C chemokine binding;GO:0002548 monocyte chemotaxis | Up |
| LOC644936 | actin beta pseudogene |  | Up |
| HLA-DRB1 | major histocompatibility complex, class II, DR beta 1 | GO:0002491 antigen processing and presentation of endogenous peptide antigen via MHC class II;GO:0002469 myeloid dendritic cell antigen processing and presentation;GO:0002468 dendritic cell antigen processing and presentation | Up |
| ANP32AP1 | acidic nuclear phosphoprotein 32 family member A pseudogene 1 |  | Up |
| EPC1 | enhancer of polycomb homolog 1 | GO:0043968 histone H2A acetylation;GO:1905168 positive regulation of double-strand break repair via homologous recombination;GO:0043967 histone H4 acetylation | Up |
| CTSZ | cathepsin Z | GO:0010757 negative regulation of plasminogen activation;GO:0002002 regulation of angiotensin levels in blood;GO:0002003 angiotensin maturation | Up |
| PBX2 | PBX homeobox 2 | GO:0009954 proximal/distal pattern formation;GO:0030326 embryonic limb morphogenesis;GO:0035113 embryonic appendage morphogenesis | Up |
| SLC25A24 | solute carrier family 25 member 24 | GO:0015867 ATP transport;GO:0051503 adenine nucleotide transport;GO:0015868 purine ribonucleotide transport | Up |
| CEP85L | centrosomal protein 85 like | GO:0001764 neuron migration;GO:0016477 cell migration;GO:0048870 cell motility | Up |
| TAF15 | TATA-box binding protein associated factor 15 | GO:0048255 mRNA stabilization;GO:0043489 RNA stabilization;GO:1902373 negative regulation of mRNA catabolic process | Up |
| CD69 | CD69 molecule | GO:0071466 cellular response to xenobiotic stimulus;GO:0009410 response to xenobiotic stimulus;GO:0070887 cellular response to chemical stimulus | Up |
| CXCL8 | C-X-C motif chemokine ligand 8 | GO:2000535 regulation of entry of bacterium into host cell;GO:0050930 induction of positive chemotaxis;GO:0060354 negative regulation of cell adhesion molecule production | Up |
| PTGS2 | prostaglandin-endoperoxide synthase 2 | GO:0010335 response to non-ionic osmotic stress;GO:0071471 cellular response to non-ionic osmotic stress;GO:0032227 negative regulation of synaptic transmission, dopaminergic | Up |
| CYP4B1 | cytochrome P450 family 4 subfamily B member 1 | GO:0018879 biphenyl metabolic process;GO:0042537 benzene-containing compound metabolic process;GO:0006631 fatty acid metabolic process | Up |
| CLASRP | CLK4 associating serine/arginine rich protein | GO:0008380 RNA splicing;GO:0006397 mRNA processing;GO:0016071 mRNA metabolic process | Up |
| MIR302C | microRNA 302c | GO:0032717 negative regulation of interleukin-8 production;GO:0032677 regulation of interleukin-8 production;GO:0001818 negative regulation of cytokine production | Up |
| RPL6P10 | ribosomal protein L6 pseudogene 10 |  | Up |
| EYA3 | EYA transcriptional coactivator and phosphatase 3 | GO:0016576 histone dephosphorylation;GO:1901099 negative regulation of signal transduction in absence of ligand;GO:2001240 negative regulation of extrinsic apoptotic signaling pathway in absence of ligand | Up |
| G0S2 | G0/G1 switch 2 | GO:2001238 positive regulation of extrinsic apoptotic signaling pathway;GO:0097191 extrinsic apoptotic signaling pathway;GO:0120162 positive regulation of cold-induced thermogenesis | Up |
| CSRNP1 | cysteine and serine rich nuclear protein 1 | GO:0060325 face morphogenesis;GO:0048008 platelet-derived growth factor receptor signaling pathway;GO:0060323 head morphogenesis | Up |
| NELL2 | neural EGFL like 2 | GO:0070050 neuron cellular homeostasis;GO:0009566 fertilization;GO:0060249 anatomical structure homeostasis | Up |
| RPS27P19 | ribosomal protein S27 pseudogene 19 |  | Up |
| NR4A2 | nuclear receptor subfamily 4 group A member 2 | GO:0051866 general adaptation syndrome;GO:0021538 epithalamus development;GO:0021986 habenula development | Up |
| MIR22HG | MIR22 host gene | GO:0035195 miRNA-mediated gene silencing;GO:0035194 post-transcriptional gene silencing by RNA;GO:0016441 post-transcriptional gene silencing | Up |
| FOSB | FosB proto-oncogene, AP-1 transcription factor subunit | GO:0051412 response to corticosterone;GO:0014072 response to isoquinoline alkaloid;GO:0043278 response to morphine | Up |
| FAM153CP | protein FAM153C |  | Up |
| DUSP2 | dual specificity phosphatase 2 | GO:0001706 endoderm formation;GO:0007492 endoderm development;GO:0035335 peptidyl-tyrosine dephosphorylation | Up |
| CCL4L1 | C-C motif chemokine ligand 4 like 1 | GO:0048245 eosinophil chemotaxis;GO:0072677 eosinophil migration;GO:0002548 monocyte chemotaxis | Up |
| CXCL1 | C-X-C motif chemokine ligand 1 | GO:0061844 antimicrobial humoral immune response mediated by antimicrobial peptide;GO:0030593 neutrophil chemotaxis;GO:0070098 chemokine-mediated signaling pathway | Up |
| FAM118A | family with sequence similarity 118 member A |  | Up |
| JCHAIN | joining chain of multimeric IgA and IgM | GO:0060267 positive regulation of respiratory burst;GO:0003094 glomerular filtration;GO:0060263 regulation of respiratory burst | Up |
| PRKCQ-AS1 | PRKCQ antisense RNA 1 |  | Up |
| ASAP1-IT1 | ASAP1 intronic transcript 1 |  | Up |
| OSM | oncostatin M | GO:0038165 oncostatin-M-mediated signaling pathway;GO:1902036 regulation of hematopoietic stem cell differentiation;GO:0032740 positive regulation of interleukin-17 production | Up |
| MAL | mal, T cell differentiation protein | GO:0098737 protein insertion into plasma membrane;GO:0001766 membrane raft polarization;GO:0002175 protein localization to paranode region of axon | Up |
| LRRN3 | leucine rich repeat neuronal 3 | GO:0051965 positive regulation of synapse assembly;GO:0051963 regulation of synapse assembly;GO:1901890 positive regulation of cell junction assembly | Up |
| CCL3L1 | C-C motif chemokine ligand 3 like 1 | GO:0002548 monocyte chemotaxis;GO:0048247 lymphocyte chemotaxis;GO:0072676 lymphocyte migration | Up |
| TNFRSF17 | TNF receptor superfamily member 17 | GO:0002260 lymphocyte homeostasis;GO:0033209 tumor necrosis factor-mediated signaling pathway;GO:0001776 leukocyte homeostasis | Up |
| HBG1 | hemoglobin subunit gamma 1 | GO:0015671 oxygen transport;GO:0015669 gas transport;GO:0042744 hydrogen peroxide catabolic process | Up |
| CCL3L3 | C-C motif chemokine ligand 3 like 3 | GO:0002548 monocyte chemotaxis;GO:0048247 lymphocyte chemotaxis;GO:0072676 lymphocyte migration | Up |
| HBG2 | hemoglobin subunit gamma 2 | GO:0015671 oxygen transport;GO:0015669 gas transport;GO:0042744 hydrogen peroxide catabolic process | Up |
| MYOM2 | myomesin 2 | GO:0045214 sarcomere organization;GO:0030239 myofibril assembly;GO:0055002 striated muscle cell development | Up |
